# Tumors and Abscesses, Causes and Treatment

**Published:** 1876-10

**Authors:** W. H. Atkinson


### DENTAL REGISTER.

VOL. XXX.] OCTOBER, 1876. [NO. 10.

### TUMORS AND ABSCESSES, CAUSES AND TREATMENT.

BY W. H. ATKINSON.

*Read before the Brooklyn Dental Society, June, 12 1876.*

Regular inflow of pabulum to the territories in which nutrition is effected lays the first step to healthy appropriation in the various degrees of fulfilment of the demand of the needy territories. Flow of currents of pabulum, retardation of flow, deflection of flow and confluence, of coinciding streams of energy and pabulum in equable movements constitute the morphological changes requisite to keep the machinery of nutrient function in good working order. Undue expression of flow, deflection of current or retardation of energy or pabulum, become arrest of nutrition at the point of expression or obstruction of these normal movements of morphological change. Thus arrest becomes obstruction, flow becomes flood and physiological metamorphosis becomes separatory action in place of combinatory procession of the elemental constituencies of tissues. And now we have a pathological instead of a physiological metamorphosis of energy and pabulum, resulting in simple exudate, tumor, tubercle, abscess or gangrene.

We are called upon to-night to make a "lay out" of the subject of "Tumors and Abscesses, causes and treatment," to serve as a target for the sharp intelligence of the members to shoot at.

*Abs*, from, and *cedo*, I go, give us the roots of our word abscess.

In works on pathology the contents of the abscess running from a rupture or opening, seem to be the underthought in the adoption and use of this word.

Without quarreling with the use that has been made of the term, I wish to exorcise its darkness by a justification of the primal idea by a delineation of the facts of the history of typical abscess.

The contents of the simplest form of abscess is air, or gas. When this is mechanically produced in a healthy body, the abscess cures by diffusion and dispersion. When pathologically produced by dissociation of the gaseous elements of cells, they are torn asunder by the production of the gas and made to go from each other, being mechanically separated by the pressure of the gas or air.

In case a single intercellular space, or only a few spaces be involved, in a tolerably fair constitution, the abscess cures by spontaneous dispersion of the gas; but in weak constitutions where a considerable number of cells have their interspaces involved in the production of the gas, another step in the departure from normal function takes place, by exudation from adjoining capillary walls, or ruptured cell walls, which exudate, absorbs the gas, either by mechanical or chemical affinity. When the absorption is purely mechanical, resorption of the exudate, and dispersion of the gas is easily induced by active exercise or local manipulation, both of which accelerate the circulation, prevent further exudation and facilitate diffusion and resorption; but when the absorption of the gas is chemical, a sort of fermentative process deteriorates the contents of the abscess, by inciting dissociation of the elements of the extravasated pabulum.

The dissociation and new association of elements in the exudate, have many measures of activity in the degree of energy and the time consumed in the abscessing process. These range from the coldest and slowest to the hottest and most rapid development of the process of absorbing territories of extravasated fluid and corpuscular blood.

Where the molecular changes are slow and all the bonds of affinity satisfied by saturation of equivalent bonds, we have the so called white swellings, or cold abscesses, as examples of this form of retrograde molecular metamorphosis.

Where the changes are rapid, and some (many) bonds of energy are set free for lack of nearness of equivalent bonds, we have hot, acute abscesses formed of the so called phlegmonous type, attended with constitutional disturbances.

The rootal sense of tumor involves the idea of swelling or enlargement. Simple enlargements of the normal tissues are called sarcous tumors. When a line of separation is set up between the normal and abnormal tissues by the formation of an adventitious membrane, which becomes the covering or sac, that holds the involved structure, we classify them then as encysted tumors, and when these have soft interiors, or fluctuating contents they present the example of an ideal abscess.

It will be perceived that all abscesses are tumors, but all tumors are not necessarily abscesses, according to the definition just given.

Whatever interferes with the normal nutrition of the tissues, which consists in an equable flow and reflow of pabulum and debris to and from the part, may be set down as a cause or antecedent condition, necessary to the formation of a tumor.

Excess of supply of pabulum is the simplest form by which tumors are produced, and is usually denominated hypertrophy. There are two forms of simple hypertrophy; the first is increase of cells in number, of normal size and function; the second is increase in size of normal cells, with corresponding increase of functional activity. Tumors thus produced are nearly always of a benign character, and are harmless, except where they, by their size, encroach upon the territory of important tissues, the function of which they mechanically disturb. The character and degree of the disturbance will give us the measure of the threatened danger.

Just at this point, it becomes us to be very careful in our observations and speculations upon the deflections of functions that constitute the degrees and lines of demarcation between benign and malign manifestations of function.

For my part I am fully convinced that all forms of malignant nutrient actions have their origin in a simple minus expression of functional energy, therefore we may conclude that any departure from normal activity, if continued, may result ultimately in greater and greater deterioration, and finally, dissolution of the part, or the entire body.

As a rule, tumors formed of analagous tissues, may be regarded as non-malignant, while those formed of heterologous tissues must be looked upon as threatning and dangerous, and demanding early extirpation wherever possible.

A rational management and treatment will now suggest itself to all who understand what has already been stated.

As all growths depend upon the supply of pabulum, it is evident that the regulation of that supply by increase, diminution or arrestation, will constitute certainty of control and efficiency of management requisite to perfect success. The details of application of these principles, involve the dynamic, physical, psychical and social relations of the case, and will be successful in the ratio of the clearness of the understanding, and the persistency of him having the case in charge and the compatible coincidence of the patient.

Extirpation of morbid growths is effected in various ways. Two principal methods of extirpation present themselves; the first, physiological, the second, mechanical.

The physiological method consists in starvation, which may be effected rapidly or gradually. The mechanical extirpation is effected surgically, by ligature, knife and cautery.

Many tumors are self-extirpative by virtue of encroachment upon the channel of supply of pabulum so as to favor starvation.

Starvation has two modes of expression that may be denominated the wet and the dry modes. Dessication, shrivelling up and gradually wasting, as in warts, or shrinking and falling off bodily, as in pedunculated moles, are examples of cure by starvation.

Another mode of starvation is where the size of the tumor has so pressed upon the vessels of supply and departure, as to arrest the circulation in the tumor.

At this point of stasis of the circulation, a molecular change or fermentative process is set up by reason of the warmth favoring the retrograde metamorphosis that first coagulates, and then refluidifies the juices and soft solids of the interior of the tumor by an abscessing process that gradually bursts the tumor, discharging its contents, presenting an ulcerous chasm upon the site of the former morbid growth which ultimately cicatrizes, thus ridding the patient of his malady without professional assistance, by the unaided efforts of nature.

Whenever we attempt to relieve a patient of a morbid growth, benign or malignant, the effort should be made to go beyond the line of demarcation between healthy and morbid tissue, at the risk of losing an unnecessary amount of healthy tissue, in preference to the probability of leaving any of the morbid growth.

In all cases where at all possible, the flaps of skin should be brought accurately together, by coaptation without tension, so as to secure union by first intention, with the least possible amount of scar tissue. Whenever we cannot secure enough skin to close the entire chasm, we should study the case well, so as to have the cicatrice come over the site of the least important tissues and organs, in order that the original function of the part may be as little influenced as possible by subsequent cicatricial contraction.

With these directions, and a scrupulous adherence to all that ministers to the hygienic conditions of the patient, the principal of which is physiological rest of the part involved in the operation, we may trust the issues of the case in the hands of any intelligent practitioner.

